# Supplementary material for: Dogs as Sentinels for Flavivirus Exposure in Urban, Peri-Urban and Rural Hanoi, Vietnam
Source: Viruses. 2021 Mar 19;13(3):507. doi: 10.3390/v13030507 (PMC8003331; doi:10.3390/v13030507)
Supplement: Supplementary file 1 [file viruses-13-00507-s001.pdf]

Household questionnaire

INTRODUCTION

Good morning / Good afternoon. My name is _________________ and I work for the International Livestock Research Institute (ILRI), which is based in Hanoi. We are conducting a study on how the presence of mosquitoes is affected by the keeping of livestock in urban environments. This study has three parts: 1) a survey to help understand the benefits and risks of urban livestock keeping and the awareness on mosquito-borne diseases; 2) to collect mosquitoes and investigate if the mosquitoes are infected with flaviviruses and, 3) to take blood samples from the livestock at household. We are visiting you because your household has been selected randomly to participate in the study titled "**The association between livestock keeping and mosquito-borne diseases in urban Hanoi**". If you accept to participate in this study, we will ask you a number of questions related to your household, knowledge and practice of livestock keeping and awareness about mosquito-borne diseases. We will also request you to get the consent to place mosquito traps on your land and take animal's blood for further laboratory analysis.

Potential risks

Your participation in this evaluation has no physical risk. We maintain strict control of all the information gained.

Potential benefits

This is an important study to control the burden of neglected tropical diseases such as dengue fever in the community.

Confidentiality

The research team promises to respect privacy and confidentiality of your information. This information will only be shared among our research team members, but we will remove all names so that no one will be able to trace back the information to you. If you voluntarily participate in this study, you may withdraw at any time without any consequences of any kind. You may also use the option of removing your data from the study. You may also refuse to answer any questions you don't want to answer and still remain in the study. The investigator may withdraw you from this research if circumstances arise that warrant doing so. You are not waiving any legal claims, rights or remedies because of your participation in this research study.

If you have any questions now or later you are welcome to call the researchers:

- **Dr Johanna Lindahl** 01675340452 (English)
- **Mr. Nguyen Tien Thang** 0981582712 (Vietnamese)If you have questions

regarding your rights as a research participant, contact:

Institutional Review Board, Room 103 Building A – Hanoi University of Public Health. No 1A, Duc Thang str, Duc Thang ward, Bac Tu Liem district, HaNoi
Tel: 024 6266 3024. Email: irb@huph.edu.vn

Questionnaire ID ☐☐ ☐☐☐

ID consists of 5 letters. The first two is district code, 3 remainings are household ID.

District code: **01** – Chuong My, **02** – Dan Phuong, **03** – Bac Tu Liem, **04** – Ha Dong

05 – Ba Dinh, **06 –** Cau Giay

No	A. General information			Note
A1	Gender	Male<br>Female	1<br>2	
A2	Age	_________		
A3	Highest level of education	College/university<br>High school<br>Secondary school<br>Primary school<br>No education	4<br>3<br>2<br>1<br>0	
A4	Marriage status	Single<br>Married<br>Separated/Divorced<br>Widowed<br>Others: …………..	1<br>2<br>3<br>4<br>99	
A5	Main occupation	Officer<br>Farmer<br>Unemployed<br>Retired<br>Others: …………..	1<br>2<br>3<br>4<br>99	
A6	Number of people in the household	_______________		
A7	Number of children under 15 in the household	_______________		
A8	Sources of water using in your family	Tap water<br>Rain water	1<br>2	

	(You can choose more than 1 option)	Rivers/lakes nearby<br>Well<br>Others: ……………......................................	3<br>4<br>5	
A9	Type of toilet/sanitation system in your family			
A10	Average income per month of your family in total	_______________ VND		
**No**	**B. Livestock information**			**Note**
B0	Is your family keeping livestock?	Yes<br>No	1<br>0	**Go to B2**
B1	Why do you not keep livestock?<br>(for household with no livestock)	Lack of space<br>No time<br>Not interested<br>No/limited knowledge<br>Concern about hygiene and sanitation<br>Others:	1<br>2<br>3<br>4<br>5<br>99	**Go to B10**
B2	Number of livestock last year	Pig ____________________<br>Goat ____________________<br>Chicken ____________________<br>Cattle _____________________<br>Duck ____________________<br>Goose ____________________<br>Others: ____________________<br>____________________		
B3	Number of livestock this year	Pig ____________________<br>Goat ____________________<br>Chicken ____________________<br>Cattle _____________________<br>Duck ____________________<br>Goose ____________________<br>Others: ____________________<br>____________________		

B4	Type of keeping livestock	Indoor<br>Tied up, partly outdoor<br>Fenced/shed in outdoor<br>Free roaming<br>Others:	1<br>2<br>3<br>4<br>99	
B5	Who is mainly responsible for keeping livestock in your home	Grandfather/grandmother<br>Husband<br>Wife<br>Children<br>Others:	1<br>2<br>3<br>4<br>99	
B6	Have your livestock get any diseases last year?	Yes<br>No	1<br>0	 **Go to B8**
B7	If yes, can you describe the symptoms or name the diseases?	___________________________________		
B8	How do you usually treat when the livestock got diseases?	___________________________________		
B9	Why do you keep livestock?<br>(Benefits)<br>***(You can choose more than 1 option)***	Provide livelihood options<br>Reduce cost and time of transportation<br>Food production<br>Recycling animal waste<br>Provide the education facilities<br>Poverty alleviation<br>Income generation<br>Pleasures<br>Others:	1<br>2<br>3<br>4<br>5<br>6<br>7<br>8<br>99	
B10	Problems and risks of livestock keeping for all households	Unpleasant smells and noises<br>Contamination in water resource<br>Affect the environment/ecosystem<br>Animal health problems	1<br>2<br>3<br>4	

		Feed supply<br>Theft<br>Inappropriate waste/manure management<br>Bring the diseases to human<br>Low production<br>Others:	5<br>6<br>7<br>8<br>9<br>99	
B11	Do you have any pets or other animals in your household?	Cats________<br>Dogs________<br>Songbird_______<br>Other pets___________		
B12	Do you know any disease that humans can get from animals?<br>***(You can choose more than 1 option)***	Don't know any<br>Rabies<br>Japanese encephalitis<br>Tuberculosis<br>Brucellosis<br>Streptococcus suis<br>Other_______________	0<br>1<br>2<br>3<br>4<br>5<br>99	
**STT**	**C. Awareness about mosquito-borne diseases**			**Note**
C1	Have you heard about diseases being transmitted from mosquitoes to humans	Yes<br>No	1<br>0	Go to C3
C2	If yes, can you list the disease(s) that transmitted from mosquitoes<br>***(You can choose more than 1 option)***	Dengue fever<br>Japanese Encephalitis<br>Zika<br>Malaria<br>Filariasis<br>Others:	1<br>2<br>3<br>4<br>5<br>99	
C3	Can you list breeding sites of mosquitoes?<br>***(You can choose more than 1 option)***	Don't know<br>Clean water<br>Drain/polluted water<br>Stagnant water containers	1<br>2<br>3<br>4	

		Tires<br>Water tanks<br>Vase<br>Bonsai rockery<br>Others:	5<br>6<br>7<br>8<br>99	
C4	Can you list the risk factors for getting mosquito-borne diseases?<br>***(You can choose more than 1 option)***	Don’t know any<br>Warm and humid season<br>High population density<br>Stagnant water<br>Livestock keeping<br>Others:	0<br>1<br>2<br>3<br>4<br>99	
C5	Can you list the symptoms while getting mosquito-borne diseases?<br>***(You can choose more than 1 option)***	Don’t know any<br>High fever<br>Muscle pains<br>Nausea/vomiting<br>Severe headache<br>Rash<br>Bleeding<br>Others:	0<br>1<br>2<br>3<br>4<br>5<br>6<br>99	
C6	In which season mosquito-borne diseases are highest?	Rainy season<br>Dry season<br>Same	1<br>2<br>3	
C7	Can you list the ways to prevent yourself from getting mosquito-borne diseases?<br>***(You can choose more than 1 option)***	Don’t know any<br>Screening of doors/windows<br>Mosquito repellent creams/ liquid<br>Mosquito nets<br>Electric rackets<br>Mosquito coils / Incense sticks<br>Long sleeves<br>Keep lids on water tanks<br>Chemical in water containers	0<br>1<br>2<br>3<br>4<br>5<br>6<br>7<br>8	

		Anti-mosquito products (e.g. insecticides)<br>Eliminate breeding sites<br>Using fish in water containers<br>Others:	9<br>10<br>11<br>99	
C8	Which personal protection do you use to prevent mosquito-borne diseases?<br>***(You can choose more than 1 option)***	Don't use any measures<br>Screening of windows/doors<br>Mosquito repellent creams/ liquid<br>Mosquito nets<br>Electric rackets<br>Mosquito coils / Incense sticks<br>Long sleeves<br>Keep lids on water tanks<br>Chemical in water containers<br>Anti-mosquito products (e.g. insecticides)<br>Eliminate breeding sites<br>Using fish in water containers<br>Others:	0<br>1<br>2<br>3<br>4<br>5<br>6<br>7<br>8<br>9<br>10<br>11<br>99	**Go to C10**
C9	Frequency of using personal protection to prevent mosquito-borne diseases?	Only at night<br>Only during the day<br>Throughout 24h<br>Few days a week<br>Others:	1<br>2<br>3<br>4<br>99	
C10	What are the sources of the information you have heard about the mosquito-borne diseases?<br>***(You can choose more than 1 option)***	Never heard<br>TV<br>Broadcast<br>Loudspeaker<br>Internet<br>Communication materials<br>Health staffs<br>Friends<br>School	0<br>1<br>2<br>3<br>4<br>5<br>6<br>7<br>8	

		Other :……………....................	99	
C11	Which sources of information you like most?<br>***(You can choose more than 1 option)***	TV<br>Broadcast<br>Loudspeaker<br>Internet<br>Communication materials<br>Health staffs<br>Friends<br>School<br>Others :……………............................	1<br>2<br>3<br>4<br>5<br>6<br>7<br>8<br>99	
C12	Have you or your family members ever get mosquito-borne diseases during the last 2 years?	Yes<br>No	1<br>0	**End**
C13	Which disease(s) has/have been diagnosed?	Dengue fever<br>Japanese Encephalitis<br>Malaria<br>No disease was diagnosed<br>Others :……………............................	1<br>2<br>3<br>4<br>99	
C14	Source of treatments for that disease	Public medical system<br>Private practitioner<br>Self – medication<br>No treatment<br>Others :……………............................	1<br>2<br>3<br>0<br>99	

Sample collection sheet for dogs

Id on the sample<br>Survey code + D1-5)	**Age**	**Sex**	**Breed**	**Any symptoms in this dog the last 2 months?**	**How is this dog kept?**<br>Like the family, follows 0<br>Indoor 1<br>Tied up, partly outdoor 2<br>Fenced/shed in outdoor 3<br>Free roaming 4<br>Others: 99	**When (which year) was the dog last vaccinated for rabies?**<br>**Never vaccinated=0**<br>**Can't remember 99**
